# Supplementary material for: Transcriptomic Profile of Genes Regulating the Structural Organization of Porcine Atrial Cardiomyocytes during Primary In Vitro Culture
Source: Genes (Basel). 2022 Jul 5;13(7):1205. doi: 10.3390/genes13071205 (PMC9319992; doi:10.3390/genes13071205)
Supplement: Supplementary file 1 [file genes-13-01205-s001.zip › genes-1795594-supplementary.pdf]

Supplementary Table S1.The list of differentially expressed genes of interest analyzed in study. Quantitative data are presented as LogFC. All of the presented sample means were deemed to be statistically significant ($p < 0.05$).

**RA**						Gene symbol	**RAA**					
**Microarray**			**RT-qPCR**				**Microarray**			**RT-qPCR**		
7D	15D	30D	7D	15D	30D		7D	15D	30D	7D	15D	30D
6,253242	5,853737	5,536136	8,391706	7,431329	8,006441	*ITGA8*	4,787222	6,306512	6,561438	3,350128	6,095644	8,224495
4,76775	5,761723	5,919576	9,441706	10,31633	10,84144	*TNC*	5,982169	6,268045	5,853354	10,37513	9,410644	9,594495
5,707601	5,924523	6,043659	3,476706	4,166329	4,626441	*FGF7*	4,673334	3,999197	4,786114	3,085128	1,545644	4,534495
4,901422	5,152612	4,723275	4,281706	5,151329	4,266441	*COL12A1*	5,00208	5,071971	4,863656	4,695128	4,285644	5,699495
2,969907	2,87327	1,633909	1,086706	1,796329	0,436441	*KIF23*	4,993154	3,372186	0,128094	3,445128	1,485644	0,769495
2,856535	2,94045	2,627044	0,676706	0,496329	0,496441	*FLNB*	2,448947	2,841917	2,475555	0,290128	0,250644	1,314495
3,340376	3,374572	2,948261	3,591706	4,601329	4,611441	*COL5A2*	1,494016	1,975676	3,004599	1,830128	2,080644	4,304495
1,575923	2,072305	1,853256	0,741706	2,376329	0,251441	*COL8A1*	2,458097	3,635923	4,017934	0,515128	2,075644	3,134495
3,131964	3,064496	3,317055	0,476706	0,936329	1,171441	*MYO5A*	3,478163	3,528242	3,359156	1,625128	0,595644	0,714495
2,259761	2,56381	2,082945	2,046706	2,521329	1,461441	*TRAM1*	2,556084	2,434098	2,244343	1,025128	0,870644	1,854495
2,753255	2,521996	2,190241	2,331706	2,391329	1,761441	*TRAM2*	2,630927	2,405767	2,591958	2,670128	1,495644	2,089495
-7,88478	-7,66408	-7,75335	-6,59329	-5,88867	-8,89856	*DMD*	-7,22937	-7,12063	-7,21656	-5,37487	-8,25436	-6,2005
-6,07981	-5,47965	-6,7634	-8,48829	-7,80867	-10,1786	*ABLIM1*	-4,27699	-4,43479	-6,56139	-7,74987	-7,83436	-13,1555
-5,19431	-6,17242	-6,59799	-7,01829	-5,74867	-6,36356	*CASQ2*	-4,33827	-5,71231	-7,09292	-6,80487	-7,40436	-9,9505

-3,25689	-3,03953	-2,13379	-6,92329	-5,33867	-2,85856	*TMOD1*	-3,61724	-2,99637	-2,43032	-7,85487	-4,65936	-4,6705
-2,68465	-2,75528	-2,63948	-8,38829	-6,61867	-6,63856	*XIRP1*	-2,54262	-3,15319	-3,62817	-4,94987	-6,07936	-6,3855
-2,5972	-3,3626	-3,30264	-6,48329	-6,84367	-5,06356	*PHACTR1*	-6,3854	-6,60626	-5,65683	-5,25487	-5,99436	-4,6655
-3,4341	-3,42236	-3,80538	-8,67329	-8,44367	-10,5136	*MATN2*	-3,47929	-4,50319	-4,39175	-7,41487	-10,1044	-8,1305
-2,97284	-2,25717	-3,00174	-6,98329	-5,15367	-6,13356	*VWF*	-3,52197	-2,86318	-3,5366	-6,21987	-5,48936	-3,8005
-2,51456	-2,31502	-2,34237	-6,90829	-7,68867	-8,15856	*ITGB6*	-2,95086	-3,04253	-2,75749	-7,36987	-7,23936	-5,4505

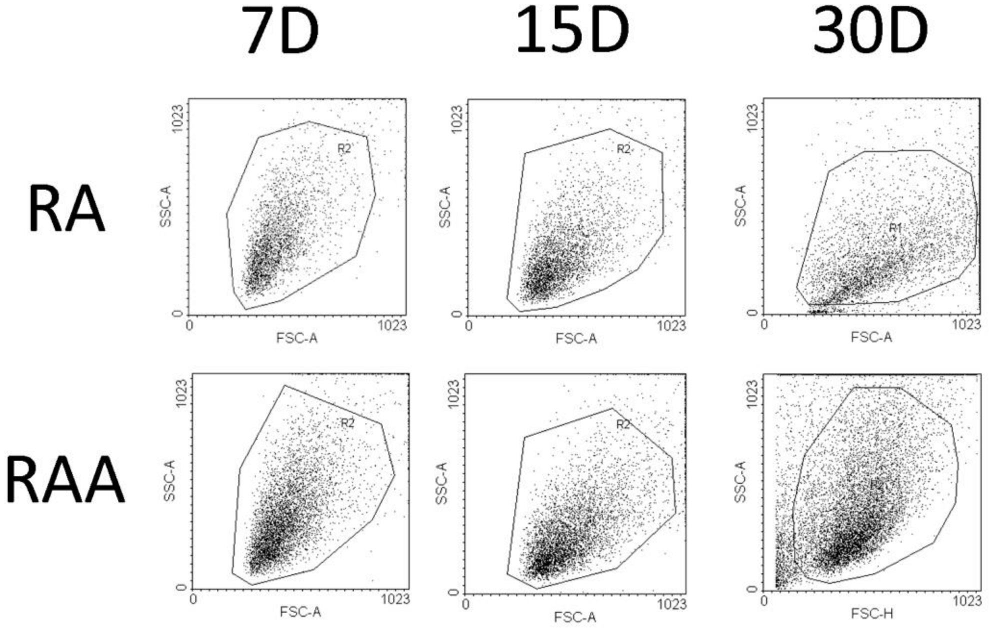

Figure S1: The flow cytometry gating strategy.

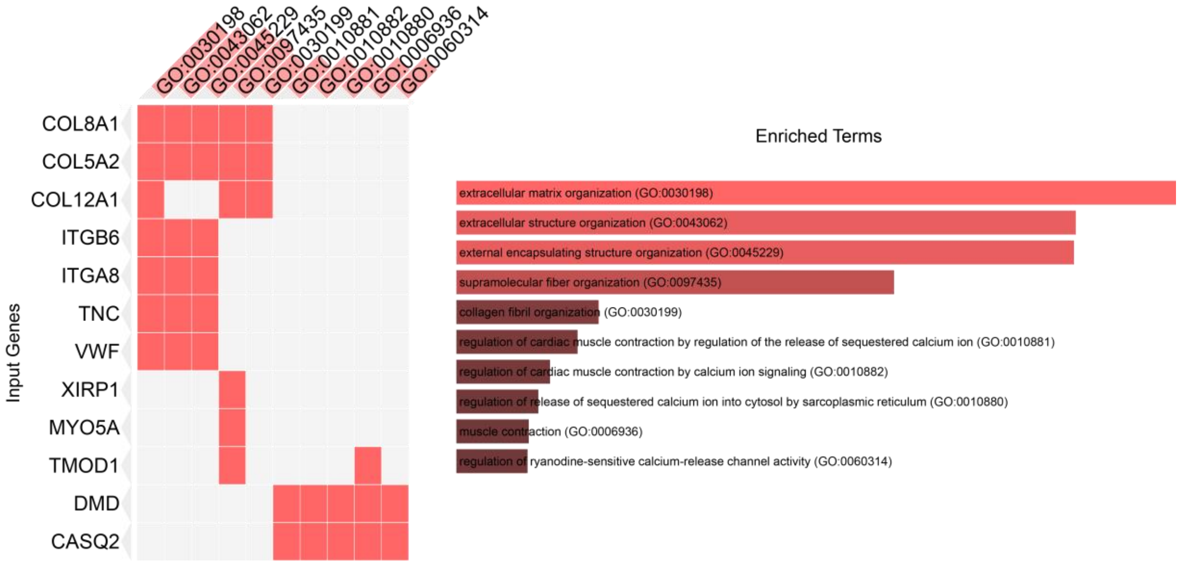

Figure S2: Enrichment analysis. In the clustergram, enriched GO terms are the columns, input genes are the rows, and cells in the matrix indicate if a gene is associated with a term.
